# Trading between healthy food, alcohol and physical activity behaviours

**DOI:** 10.1186/1471-2458-14-1231

**Published:** 2014-11-27

**Authors:** Emma L Giles, Mary Brennan

**Affiliations:** Institute of Health and Society, Newcastle University, Baddiley-Clark Building, Newcastle upon Tyne, Tyne and Wear, NE2 4AX UK; The University of Edinburgh, Business School, 29 Buccleuch Place, Edinburgh, EH8 9JS UK

**Keywords:** Lifestyle behaviours, Food, Alcohol, Physical activity, Young adults, Trade off behaviours

## Abstract

**Background:**

While recent lifestyle studies have explored the role that food, alcohol or physical activity have on health and wellbeing, few have explored the interplay between these behaviours and the impact this has on a healthy lifestyle. Given the long term health advantages associated with leading healthier lifestyles, this study seeks to: 1) explore the interplay between the food, alcohol and physical activity behaviours of young adults (aged 19–26 years) in the North East of England; 2) explore the trade-offs young adults make between their food, alcohol and physical activity behaviours; and 3) recognise the positive and negative associations between the three behaviours.

**Methods:**

Qualitative self-reported lifestyle diaries and in-depth interviews were conducted with 50 young adults from the North East of England between February and June 2008. Qualitative thematic analysis was undertaken using Nvivo QSR software, and diary coding using Windiets software.

**Results:**

Young adults who attempt to achieve a ‘healthy lifestyle’ make trade-offs between the food and alcohol they consume, and the amounts of physical activity they undertake. There are negative reasons and positive consequences associated with these trade-offs. Young adults recognise the consequences of their behaviours and as a result are prepared to undertake healthy behaviours to compensate for unhealthy behaviours. They prefer certain strategies to promote healthier behaviours over others, in particular those that relate to personalised advice and support, more affordable ways to be healthier and easily-accessed advice from a range of media sources.

**Conclusions:**

Young adults seek to compensate unhealthy behaviours (e.g. binge drinking) with healthy behaviours (e.g. physical activity). Creative solutions may be required to tackle these trade-offs and promote a balance across the food, alcohol and physical activity behaviours of this age group. Solutions that may be effective with this age group include environmental changes (e.g. green spaces and increasing the price of alcohol) designed to encourage and facilitate young people making healthier choices and improving their access to, and lowering the price of, healthy food products. Solutions must recognise these trade-offs and in particular, the strong reluctance of young adults to alter their higher-than-recommended levels of alcohol consumption.

## Background

A healthy lifestyle has been shown to be advantageous for many reasons, such as weight regulation [1], happiness and wellbeing [2], and to reduce the personal, societal, and economic consequences of lifestyle-related illness and disease [3]. While recent lifestyle studies have begun to explore the complex relationship between food, alcohol and physical activity and how this relates to weight management, health status and behavioural change [4–7], there has been limited attention paid to how these three behaviours interact with each other in determining an individual’s daily energy balance (energy consumed and energy expended) and how such interactions can influence an individual’s overall weight, health status and lifestyle choices [8]. Previous research has explored with young adolescents how their food and physical activity lifestyle behaviours influence their energy balance and body composition, and found that active adolescents do not always consume healthier diets [9]. Building on these findings, this study explores similar issues with young adults, and focuses not only on dietary composition and physical activity, but on the range of wider moderating factors which impact on them leading and maintaining a healthy and active lifestyle.

In particular, adults should undertake 150 minutes of moderate activity each week; consume five portions of fruits and vegetables per day (in addition to other macronutrient intakes) and consume no more than two to three, and three to four, units of alcohol per day respectively for women and men. These governmental recommendations are provided for physical activity, dietary intake and alcohol consumption [10–22], yet studies have shown that few young adults in England meet these recommendations.

For physical activity, in 2012, 83% of young men aged 16–24 years met physical activity recommendations, but only 57% of young women in this age group. Additionally, 10% of men and 28% of women aged 16–24 years had low physical activity levels [23]. Although these figures are an increase on 2008 levels, when only 53% of young men and 35% of young women in this age group were estimated to be meeting the recommended physical activity guidelines [24–29]. In terms of alcohol consumption, in 2012, 27% of young men and 19% of young women aged 16–24 years drank more than eight and six units respectively, on their heaviest drinking day in the past week when surveyed. These unit intakes constitute double the recommended unit alcohol intakes for any given day and is known as binge drinking [23, 30]. Additionally, for fruit and vegetable consumption, only 15% of young men and 20% of young women aged 16–24 years said that they consumed five or more portions per day in 2012 [23]. Despite some recent improvements observed between the 2000/2001 and 2008/2009 National Diet & Nutrition Survey [24, 25, 28, 29, 31], concerns remain about the nutritionally poor dietary intakes of young adults in England, particularly in relation to these low levels of reported fruit and vegetable consumption and high levels of reported consumption of unhealthy snacks, convenience foods and sugary soft drink consumption. Certainly, these unhealthy behaviours are not atypical of other young adults, with studies with German students reporting similar levels of binge drinking, and low levels of fruit and vegetable consumption and physical activity [32, 33].

This is particularly worrying for young adults transitioning from childhood to adulthood, as there is evidence to indicate that if energy imbalances occur in late childhood and early adulthood the resulting weight gain can remain into full adulthood through a process termed “*tracking”*[34]. Together this evidence paints a worrying picture of nutritionally-poor dietary intakes, excessive alcohol consumption and a lack of physical activity amongst some young adults and may go some way to explaining the upward BMI trend observed in particular amongst young males between 1993 and 2010 [35, 36].

While some encouraging recent improvements have been observed across all three of these behaviours in young adults [31], it is not yet clear whether there is any interplay between the food, alcohol and physical activity behaviours of young adults, and what, if anything, this may mean for the healthiness of their lifestyles. Limited research, from the UK, has explored this potential interplay with young adults, with studies often focusing on one or two of these behaviours, rather than all three [37–39]. Where research does focus on the interplay between all three behaviours, it does not explore this issue with a UK-based sample of young adults [40–44]. Therefore, this study seeks to make a novel contribution to the extant literature by qualitatively examining the interplay between the food, alcohol and physical activity behaviours of young adults from England, and the daily trade-offs that these young adults make between these behaviours in their attempts to achieve and maintain a “balanced, healthy” lifestyle. This is important, given that cultures have their own specific norms and behavioural practices which may not always be transferable to other contexts.

The objectives are threefold and are designed to explore and capture: 1) the interplay between the food, alcohol and physical activity behaviours of young adults (aged 19–26 years) in the North East of England; 2) the trade-offs young adults make between their food, alcohol and physical activity behaviours; and 3) the positive links between, and negative associations with, the food, alcohol and physical activity behaviours of young adults.

## Methods

This exploratory study involved the use of self-reported lifestyle diaries and in-depth interviews to explore the lifestyle behaviours of young adults. Ethical approval was granted by Newcastle University Research Ethics Committee; all participants provided written consent before participating; and were verbally informed in the interviews of the informed consent process, data confidentiality and anonymity issues. By participating, they agreed to the terms. All participants received a £10 voucher to nominally reimburse them for their time and travel expenses for the interviews, and £10 per diary (total of £40).

### Participant characteristics

Initially 18–25 years olds were targeted. This age range was chosen given limited previous research with this age group and that it is categorised as a period of ‘emerging adulthood’ when young adults take more responsibility for their behaviours [45, 46]. Participants were recruited via posters and recruitment notices in community centres, supermarkets, and libraries, and through emails and internal web notice boards in Newcastle University and Newcastle City Council. Participants self-selected themselves for this study, and were not chosen by the researcher. In addition, snowballing techniques were also used with those initially recruited, which is consistent with sampling approaches used in qualitative research [47]. Whilst 18–25 year olds were initially sought for the study (for ethical reasons and because the legal age in the UK (United Kingdom) to consume alcohol is 18 years), one participant turned 26 during the study and no 18 year olds volunteered. As a result, the final sample was made up of 19–26 year olds. The sampling approach taken was not designed to provide generalisable results [48] and we urge caution in drawing generalised conclusions from the results. However, this exploratory study does provide interesting insights into the interplay between the food alcohol and physical activity behaviours of young adults and is a useful platform from which to inform further research in this area.

In total, fifty participants took part in the diaries and interviews. This sample size was chosen in order to provide a cross-section of participants, but acknowledging resource limitations. The sample was dominated by female (60%) and student (60%) participants. Participant incomes ranged between £0 and £30,000, with 44% of the young adults having an average income of £10-19,999. Ages ranged from 19 to 26 years, and 72% reported their marital status as single. Of the sample, 80% expressed no explicit dietary requirements, with the remaining 20% declaring themselves to be either vegetarians/vegans, following a diet of low carbohydrates, fat and high protein, or following a weight-reducing diet. Whilst only a small proportion of the young adults explicitly indicated that they were following a particular diet, the interview discussions indicated that many more, while not following a particular diet, were trying to lose weight. Overall, the majority of participants (66%) were classified as falling within a healthy weight range (using self-reported height and weight data), 22% were classified as overweight and 12% as obese.

### Methods, data collection and analysis

The young adults were given a lifestyle diary to complete for four days per week, for one week per month over the course of four months (February-May 2008). They completed two four-day cycles from Wednesday to Saturday and two four-day cycles Sunday to Wednesday, to ensure at least one weekend day was included in each cycle. All participants completed all four diaries.

The diaries were self-reported, in that the participants completed paper and/or electronic versions of the diaries depending on their preference. Each diary asked participants to record all of the foods and drinks (alcoholic and non-alcoholic) they consumed each day, who they consumed them with, where and when they were consumed, the amounts consumed, cooking and preparation methods used, the brand of food or drink consumed and any additional comments. They were asked to provide portion sizes in a format that suited them (e.g. grams, serving/plate size) and were provided with examples at the front of the diary. The diaries also asked them to record all of the moderate intensity physical activity they undertook each day, who they undertook the activities with, where, for how long, an activity description and the price paid (if any). They were all asked if each day was ‘typical’ for them and to explain any atypical behaviour that may have arisen from occasions such as celebrations or holidays. General questions at the end of the diary asked about whether participants were following any specific diet, whether they took food supplements, their hours of work and/or study over the diary days, and an estimate of how much they spent on food, drink and activities during each four-day diary period.

Following preliminary analysis of the diaries using Windiets and manual coding, one to one interviews were conducted with each participant, during which the behavioural patterns outlined in the diaries were discussed, alongside other questions. These in-depth interviews were conducted with 50 participants (by ELG) in Newcastle University buildings during June 2008 with each interview lasting between 45–60 minutes. These interviews were partly based on the self-reported diaries completed by the 50 young adults prior to the interviews, to identify their food, alcohol and physical activity behaviours (see Giles et al., under review) [49]; hence why interviews were chosen so that the participants did not have to share their data with others (i.e. as they would have done in a focus group setting). The semi-structured interview guide also asked participants about their current work, study, and/or lifestyle patterns (research objectives 1 and 2), what they thought being ‘healthy’ meant for them (research objectives 1 and 2), their overall attitudes towards their food, alcohol and physical activity behaviours, if they would change their behaviours and why they would or would not, whether they would need support to change their behaviours and what this support would be, and how motivated they would be to change their behaviours given the associated costs and benefits. Questions were also asked on their food, alcohol and physical activity behaviours (research objective 3), gave the participants an opportunity to reflect on their (diary) behaviours, allowed each individual to comment on their lifestyle, and provided an opportunity to explore whether the participants were happy with their current lifestyles (research objective 3). If they indicated that they would change their behaviours, then the interviews also discussed when, how and why they would change their behaviours. Probes were used to explore issues that were not *a priori* listed in the discussion guide, but which arose during the interviews.

All interviews were audio recorded, transcribed verbatim and analysed using thematic analysis by one researcher (ELG). The transcribed interviews were coded using NVivo (QSR International) software, using both *‘in vivo’* and sociologically constructed codes, as per an inductive process. The former uses the exact words of participants to inform the coding, whilst the latter employed codes that the research team identified as the most relevant to the meaning of the data [50, 51]. The preliminary analysis and the resulting codes were checked and verified by a second researcher (MB). Representative quotations are provided in the results section below. At all points, the researchers remained objective, and did not influence the research collection and interpretation.

At the analytical stage no specific theories were chosen *a priori* to structure the data analysis process, although it is acknowledged that there are several psychological and public health theories that have been used to explore individually food, alcohol and physical activity behaviours, including the Health Belief Model [52], the Stages of Change Model [53], Self Efficacy/Social Cognitive Theory [54, 55] and the Theory of Planned Behaviour [56, 57]. The reason for not choosing one or more theories upfront to explain behaviour, was that each of the theories have their own advantages and disadvantages, and choosing one theory in advance would be presupposing particular explanations for the reported behaviours of the young adults. Additionally, none of the theories supported the holistic investigation of the interplay between the three behaviours of interest investigated in this study. Therefore, although inspiration was taken from the available theories, none were applied in full during the early analytical stages. By drawing upon the principles of grounded theory, an inductive coding process [58] was used that focused on coding the reported behaviours. This was achieved by adopting a behavioural perspective that privileged reported behaviours and the reasons given for these behaviours. It was also felt to be inappropriate to make *a priori* assumptions on the basis of the socio-economic characteristics of the participants. At all stages, we adhered to RATS guidelines (http://www.biomedcentral.com/authors/rats).

## Results

Table 1 shows that within the sample, many of the young adults did not meet recommended guidelines for food and physical activity in particular. This was calculated by taking an average of their behaviours from across their diaries [59]. It is shown that for alcohol consumption, most of the young adults met recommended guidelines based on an average of their alcohol intake from across the diary period. This however, masks the fact that nearly all of the young adults binge drank on one or more of their diary days.

**Table 1:** **Percentage of the sample meeting food, alcohol and physical activity recommendations**

***Average from four days diary data***	Food (±500 kcal/day compared to recommendations)	Alcohol (meeting or consuming less than recommended units/day)	Physical activity (meeting or exceeding recommended minutes/day)
**Men**	45%	95%	10%
**Women**	53%	100%	0
**All**	50%	98%	4%

Exploring why these recommendations were not met, identified three separate issues which will be discussed below:

1. The young adults appear to make trade-offs between food and alcohol consumed, and the physical activity undertaken when aiming to achieve a ‘healthy lifestyle’. This further highlights the interplay between the three behaviours;
2. There are both positive and negative consequences as a result of the trade-offs made, and the interplay between, the food, alcohol and physical activity of the young adults.
3. Certain strategies are preferred over others, by the young adults, to help them to be healthier across their behaviours.

### The interplay and trade-offs between food, alcohol and physical activity behaviours

What became apparent from the interview data was that few of the young adults reported meeting food, alcohol and physical activity recommendations for all three behaviours (this was underpinned by analysis of their diary data; see Giles, 2010) [13, 14, 16, 59]. They reported trading off between the three behaviours for reasons that included: looking for redemption after poor behaviour in one or more categories; trying to be healthier in some lifestyle areas; instilling some semblance of balance across these lifestyle behaviours, facilitating engagement in certain ‘prioritised’ lifestyle behaviours (i.e. binge drinking); and rewarding themselves for being ‘good’.

It was apparent that some participants viewed engaging in healthy behaviours (i.e. physical activity; increased fruit and vegetable consumption) as a form of redemption (and/or compensation) for engaging in unhealthy behaviours (i.e. binge drinking; eating fast food/confectionary):

*“I consider it it, well, you know, I would be unhealthy in drinking quite a lot on one side, I like to at least try and eat a certain amount of vegetables and cook er kind of healthily so yeah.”* [Interview 010]

*“So exercise plays a big part in me being happy with my size. So if I didn’t do it, then I probably wouldn’t eat the chocolate…”* [Interview 005]

Similarly, balancing out unhealthy behaviours with healthier behaviours in an attempt to instil a sense of balance across one’s lifestyle was evident:

*“I try and be as healthy as possible, but I know that I, you know, I have the odd cigarette occasionally, and I drink a bit. So I think it’s, it’s okay to have a little bit of something bad, though.”* [Interview 052]

Whilst this desire and attempt to achieve balance prevailed across many of the young adults, it did not go as far as including all three behaviours. Often it meant the young adults reported engaging in healthier food and/or alcohol behaviours but made no mention of physical activity, or followed healthier food and/or physical activity behaviours, with limited consideration for their alcohol behaviours. In few instances did the young adults report instilling and maintaining a sense of balance across food, alcohol *and* physical activity behaviours:

*“I think I’m pretty, er, I think I’m very, I’m very focused. You know, I … it’s an important thing for me to try to stay healthy and to keep fit and things like that. So it is a big part of my life. So, you know, in terms of my lifestyle, keeping, eating food, the right, eating the right food, and the right kind of diet, and also drinking the right kind of stuff…”* [Interview 023]

There were trade-offs between the behaviours, with some behaviours prioritised over others. Often this linked back to a lack of time to engage in healthier behaviours across all three areas:

*“Well, I do do a lot of sport, so that’s kind of a healthy thing for me, but the food I eat, er, my attitude towards that isn’t very good. I don’t like what I eat, to be honest. It’s, er, horrible stuff, but it’s quick and it’s easy.”* [Interview 050]

Finally, trade-offs were evident in that adherence to healthy recommendations in one lifestyle behaviour was used to justify a reward of a different, but unhealthier, behaviour. Thus, quite often participants who engaged in healthy food and/or physical behaviours rewarded themselves by engaging in another unhealthy behaviour (i.e. drinking alcohol; a bar of chocolate):

*“I think it’s just a treat maybe, ‘cos with going to the gym and trying to eat quite healthily, when I do go out, for an occasion, I try to, like, it’s like an occasional treat.”* [Interview 051]

This pattern of trading off between lifestyle behaviours appeared to be common across the majority of the young adults. Whilst for the most part the reasons for such trade off behaviours were due to their desire to engage in some unhealthy food, alcohol or physical activity behaviours, there was some evidence that such trade off behaviour may have some positive consequence for maintaining balance and meeting some food, alcohol and physical activity recommendations. Both the negative and positive consequences will now be discussed.

### Trade-offs: negative reasons and positive consequences

Engagement in trade-off behaviours was said to be for negative reasons when the young adults perceived themselves as having a lack of time, and paradoxically, for some, an excess of time:

*“I used to like really watch what I ate. I used to, em, I used to go jogging a lot, em, and I used to do like a lot of exercise. But then I came to Uni, and it just went of the window.”* [Interview 049]

*“Yes probably because I’ve got more free time, more flexible free time on my hands. I can afford to probably get more drunk or eat probably more lavishly throughout the day than people who are more tied to kind of nine to five jobs.”* [Interview 010]

There was also a sense of apathy amongst some of the young adults. They talked about not being bothered to follow healthy lifestyle behaviours as they did not care enough about their lifestyles and/or the potential impact their lifestyles may be having on their own personal health:

*“I’ve always taken kind of a laissez-faire attitude to, well, foods and activities.”* [Interview 008]

In some instances, the young adults also recognised additional negative consequences arising from their engagement in certain unhealthy behaviours (i.e. binge drinking) which had repercussions for other lifestyle behaviours:

*“I guess it’s just because we were drinking in the day time, like drinking in the afternoon, and like when you have a couple of pints of lager, then you don’t feel like eating a proper meal anyway…”* [Interview 017]

*“Hangover days tend to be lazy days.”* [Interview 040]

Conversely, positive outcomes were also evident the linkages that some of the young adults made between their food, alcohol and physical activity behaviours. In particular, some young adults reported engaging in certain healthy behaviours in order to be able to be an all-round healthy individual and to gain a sense of balance across their food, alcohol and physical activity lifestyle behaviours:

*“…sort of try and keep a balanced, try and do exercise every day, and like eat fresh food.”* [Interview 028]

*“But if I do the sport thing, em, and if I do coaching on a certain day, I tend to, I’ll eat more fruit on that day.”* [Interview 019]

In addition, some participants acknowledged the importance of monitoring the healthiness of their food and the amount of alcohol they are drinking as part of their attempts to improve their physical fitness:

*“I’m at the gym, em, and alcohol does impact on, you know, your, em, respiration and metabolism. So again, just again, to help myself, rather than working against myself, I try not to drink too much.”* [Interview 029]

*“…try to have a, you know, a high protein diet. That’s all tied in with going to the gym and stuff like that, because that’s, er, you know, there’s no point in going to the gym if you’re not on a high protein diet…”* [Interview 023]

Even though they monitored their behaviours, where they did not meet recommended guidelines, many of the young adults were not motivated/willing to change their behaviours so that they met recommended food, alcohol and physical activity guidelines. This was largely due to ‘laziness’:

*“Laziness I suppose. Like it’s easy, it’s lazy to just sit at your desk and have your dinner.”* [Interview 005]

*“…it takes quite a lot of effort to alter those kinds of things.”* [Interview 017]

A lack of impetus to change behaviours fed into the suggested strategies that the young adults said that they would prefer to encourage them to be healthier in their behaviours.

### Preferred strategies to promote healthier behaviours

The majority of suggested strategies that the young adults said would help them to change their behaviours, centred on making healthier behaviours more affordable. In particular, subsidising gym membership for example, or for retailers to make healthier foods more affordable in comparison to less healthy foods:

*“I’m paying for the gym… and things like that should be more, should be subsidised.”* [Interview 006]

*“Well, actually, I would like it if, em, like good food was actually cheaper.”* [Interview 017]

Providing advice and support was also a key consideration. This could be in the form of healthy recipes, personalised nutrition advice, one-to-one support, or advice from a personal gym trainer:

*“Maybe you could have healthy recipes that were really like easy and quick to make.”* [Interview 017]

*“I always thought it would be interesting, you know, to speak to a nutritionist about my diet and how I can improve it.”* [Interview 016]

*“…someone to tell me what I should be eating, what I shouldn’t be eating, and how much exercise I should do.”* [Interview 049]

*“…to have a personal trainer to tell me what to eat and how to, and what to do every day as exercise.”* [Interview 022]

The young adults also identified that a wide range of media sources could be used to provide information on healthy behaviours, including the use of the internet, television, and magazines. Being able to check health behaviour recommendations on the internet, and promoting health messages as advertisements were seen to be relevant for young adults. This includes advice in magazines that young adults are likely to read:

*“if it came out in an article on the internet”* [Interview 036]

*“…cause most people have a TV, and that way it’ll be free and because you can put adverts in between”* [Interview 006]

*“…sometimes read like a magazine, or something like that, like a fitness magazine”* [Interview 023]

## Discussion

### Food, alcohol and physical activity behaviours of young adults

Analysis of the interview and diary data found that the food, alcohol and physical activity behaviours of the young adults were inter-related and for many, were traded off between each other. They said that they try to be healthier in one area of their lifestyle (i.e. food behaviours) in order to justify being less healthy in another (e.g. alcohol consumption). Generally, many of the young adults discussed the idea that they try to be healthier in their food intake and/or physical activity behaviours to compensate for unhealthy alcohol behaviours (i.e. binge drinking). Indeed research has identified the need for behavioural interventions to target dietary intake and physical activity because of the links between the two, but without forcing young adults to change their behaviours [60].

The young adults were trading off their food and physical activity behaviours, by increasing their physical activity levels to compensate for their over consumption of ‘junk food’. Likewise if they did not enjoy participating in physical activity, they were willing to consider and/or engage in self-regulation of their diet. This is similar to previous findings, where an investigation of college student behaviour found that students reported physical activity participation to counterbalance the calories they consume from alcohol [39]. A sense of balance across the three lifestyle behaviours was not evident amongst these young adults partly because they place more value on short term gains (e.g. enjoyment from junk food, over the long term benefits of a balanced diet), and as such fail to regulate all three behaviours simultaneously for fear of losing some of the enjoyment they currently associate with eating junk food and binge drinking [61]. This is problematic in the sense that it is not clear if trading-off behaviours results in negative health outcomes, or whether healthy eating and physical activity can ‘offset’ the impact of excessive alcohol consumption. Certainly, balanced lifestyles are advocated [62] and this is probably because of the impact unhealthy behaviours can have even when supplemented with healthy behaviours [63].

Where their behaviours did not meet recommended guidelines (such as alcohol consumption), they were willing to trade-off the after-effects of unhealthy behaviours (e.g. reduced physical activity) for short term gains (e.g. excessive alcohol on a night out). This is consistent with the time perspective literature, with research finding that young adults value small immediate rewards over larger future rewards [64, 65]. This helps to explain why these young adults continue with unhealthier behaviours, because they gain immediate enjoyment from them, and which could be perceived as more valuable compared to distant health benefits that would arise if healthier behaviours were adopted. The value obtained by these young adults from their less healthy food, alcohol and physical activity behaviours was very much focused on obtaining short term gains, undertaking behaviours that were as easy as possible to follow, enjoyable to partake in, and which were also exhibited by their peers. Certainly for alcohol consumption in particular, the role of peer influence has been well established as playing an major role in encouraging excessive alcohol consumption [66]. That said even when the young adults expressed a desire to be healthier, and could identify value from doing so they were not very successful in putting the desire to be healthier into practice. Partly this was because they lacked impetus to be healthier. This lack of effort to engage in healthy behaviours has certainly been found in terms of food preparation, with research finding that young adults do not prepare their own foods on a weekly basis and which translates into increased fat intake [67]. This was certainly reflected in this study, with a ‘laissez-faire’ attitude.

Many of the young adults said that regulating their alcohol consumption was not a priority for them. In exploring the interplay between the food, alcohol and physical activity behaviours, it was found that the young adults were more likely to make healthier food and physical activity changes than alcohol changes. This highlights the trade-offs considered and/or made by young adults, and a general reluctance amongst the young adults to alter their alcohol consumption due mainly to the enjoyment they get from their current patterns of consumption, Whilst for some this would not be a problem, for others excess alcohol consumption is contributing towards an excessive energy intake [10] and has the potential to have adverse health effects when consumed in binge quantities [22, 68–72]. Certainly, for some of the young adults, excessive alcohol consumption led them in the short term to consume unhealthy foods and limited their physical activity (for example an excess of alcohol and the resulting hangover can lead to increased junk food consumption and a reduction in the quantity and quality of physical activity undertaken the following day).

### Strategies to encourage young adults to be healthier

As stated above, the option of reducing their alcohol intake was something that few of the young adults were willing to consider, regardless of demographic characteristics (e.g. employed and students). They were committed to continuing to consume alcohol, particularly in binge quantities, and were not willing to consider, nor change their alcohol consumption patterns in the foreseeable future. This was despite the fact that many recognise that binge drinking affects other lifestyle areas and can result in them eating junk food during or after a night out, or not doing any physical activity when hung-over. In general, their alcohol consumption was the least considered, least discussed, and the behaviour least open to regulation and or change by these young adults. Such a finding is similar to those found in a review of UK student drinking behaviour. Gill [21] found significant levels of binge and heavy alcohol consumption in young adults, and suggested that this age group were paying little attention to guidelines on sensible alcohol consumption. It would appear that action to reduce alcohol consumption and episodes of binge drinking is not likely to stem from conscious decisions and actions of individuals in this age range. Instead, it is likely that external environmental changes may be needed to reduce their likelihood and incidences of binge drinking. Such changes may include: minimum alcohol pricing [73]; changes to the legal drinking age; and changes to licensing laws and opening times and restrictions on alcohol advertising. In terms of environmental factors leading to increased alcohol consumption in young adults, factors such as price of drinks, venue style, and even temp of the music can impact on the amount of alcohol consumed [74]. These are wider factors that require further consideration if young adults’ alcohol intake is to be unconsciously changed – given that this research identifies it to be unlikely that conscious changes to alcohol consumption may occur in this age group.

That said, and learning from this exploratory study, any behavioural intervention may need to focus on food and physical activity, but rather than ignoring alcohol-related behaviours, focus on increasing young adults’ motivation to change their alcohol behaviours. This certainly links to the Stages of Change model [53], and suggests that these young adults may be in the action and planning stages in terms of food and physical activity, but in pre-contemplation and contemplation stages in terms of alcohol consumption. This would suggest the use of motivational interventions to have a positive impact on alcohol intake in young adults in the short term and assist them in moving into the planning and action stages of change [75, 76]. Matching an individual’s stage of change to the intervention seems to be particularly important in relation to young adults and their alcohol consumption [77].

Regarding the trade-offs, it may be useful to instil a greater sense of the importance of achieving balance across young adults’ food, alcohol and physical activity behaviours, and to do this by developing a set of tailored communications designed to clearly inform young adults of dietary, alcohol and physical activity guidelines, the personal benefits of living a balanced lifestyle and the health and other consequences associated with not achieving a healthily, balanced lifestyle. Tailoring of health communication messages has been found to be successful [78]; and such tailoring of messages for young adults should focus on: 1) what a balanced young adult’s diet can look like (including one that includes junk food, alcohol and ‘healthy’ foods); 2) how physical activity can be easily incorporated into daily routines (which they indicated they are more likely to do), such as commuting to work/college/university on foot or by bike instead of by car/public transport; and 3) how to engage in healthy, yet enjoyable, alcohol consumption. Such tailored communications would also need to highlight how the ‘good’ in certain healthy behaviours (e.g. healthy food intake) can be easily undone by the unhealthy consequences of other behaviours (such as binge drinking), and promote that young adults should strive to achieve a lifestyle underpinned by a healthy balance across all their food, alcohol and physical activity behaviours. Additionally, and in terms of promoting these tailored messages, mass media campaigns have been shown to have a modest effect on changing health behaviours [79], and certainly correlates with social marketing initiatives to change behaviours [80]. But what is important, is that the choice of media to promote these messages to young adults should be consistent with the sources that they would actually go to access information, such as the internet and fitness magazines as indicated in this research.

It is clear that simply educating these young adults by providing recommendations is insufficient if current messages are seen as confusing, viewed as irrelevant to them, not valued by them and not even ‘on their radar’ (i.e. a reluctance to alter alcohol intake) [59]. Highlighting a realistic approach to achieving a balance across the behaviours and not a ‘holier-than-thou’ approach is essential, so that young adults can relate to the recommendations and more easily comprehend how they could fit them into their lifestyles. This may mean significantly altering the way that health messages are communicated to this age group, including the wording, content and framing of such messages.

Regardless of where these young adults currently are in terms of their behaviours or where they would like to be, they need to be motivated and supported to engage consistently in healthier food, alcohol and physical behaviours. Certainly, counselling has been found to be an effective way to alter health behaviours [80], and the support of individuals such as personal trainers and nutritionists was identified as helpful sources of support by the young adults in this research. Other initiatives could include, for example, free exercise classes, reduced gym fees, and healthy lifestyle community support groups, but would require greater collaboration between industry, government and communities, at national regional and local level, to establish the most appropriate, cost effective and locally relevant reward, encouragement and support mechanisms [81].

Providing education, encouragement and support to those making incremental lifestyle changes may help in highlighting the benefits of ‘small but significant’ healthy lifestyle changes [82]. However, if they are still unwilling to voluntarily change their behaviours (e.g. alcohol intake) then enforcement, such as the proposed minimum pricing proposals for alcohol [83] (like the ban on smoking in public places [84]) may be required, coupled with environmental changes designed to support and encourage healthy lifestyles, through for example making healthier food more widely available, front of pack labelling on foods and drinks, unit labelling on alcohol, the development of novel and attractive low and non-alcoholic drink alternatives, incentives for walking/cycling to work; and an increase in facilities and green spaces to support greater engagement in physical activity [85]. Such environmental changes seek to make it easier, cheaper, more convenient and/or more likely for young adults to engage in healthier lifestyle choices.

### Limitations of this research and suggestions for future research

Before concluding our findings, it is important that the limitations of this research are highlighted. Firstly, it is worth reiterating that caution needs to be taken when interpreting these results given the convenience and snowballing sampling approaches used and the qualitative nature of the study, which may not be wholly typical of the wider 19–26 population. Secondly, the interviews (and the diary data on which the interviews were partly based) are self-reported accounts of behaviours, and so misreporting of behaviours is a possibility. Thirdly, as participants were remunerated for taking part in the study, this may have encouraged certain young adults to attend the interviews to obtain the incentive and may have resulted in them seeking to please the researcher. Similarly, those young adults who were generally interested in their food, alcohol and physical activity behaviours may have been more likely to respond to the recruitment notices, meaning that the opinions of other young adults without this general interest are missed. Lastly, whilst the interviews prompted the young adults to discuss whether or not they would change their behaviours, no support was provided to help them change their behaviours after the study. This was due to not obtaining *a priori* ethical permission to be able to offer personalised advice and guidance. That said, this in-depth qualitative study offers valuable exploratory insights into the interplay between the food, alcohol and physical activity behaviours of young adults. The depth in which the issues were explored was only possible using a qualitative approach and the results provide a useful basis from which to develop more quantitative and generalisable research instruments in the future. Future research would benefit from including objective outcome measures and use of technologies such as accelerometers, apps, and smart technologies to assess lifestyle behaviours in young adults. Further research into how young adults can be better facilitated to make healthier lifestyle changes across all three key lifestyle behaviours rather than trading between them is also strongly recommended.

## Conclusions

In conclusion, this exploratory study found that young adults seek to compensate unhealthy behaviours with healthy behaviours. In essence, they trade-off their behaviours, preferring to alter their food and activity behaviours to compensate for less-than-recommended alcohol consumption behaviours. As a result the key recommendations arising from this study are that young adults need be encouraged and facilitated to adopt a sense of balance, to avoid unhealthy trade-offs, to enjoy eating healthier, to increase their participation in physical activity and to drink alcohol in moderation. Highlighting how to achieve a balanced lifestyle, emphasising how guideline recommendations are applicable and relevant to them, and considering wider environmental changes that can better support healthier behavioural choices, are all key action areas.

Whilst this research raises more questions than it answers, it is clear that more research is needed to explore how, if at all, it is possible to better ‘market’ healthier living, and in particular healthier levels of alcohol consumption, to young adults. As such, the authors strongly recommend that further research is undertaken to explore the emerging interplay between the three behaviours, the impact of this for energy balance maintenance amongst young adults and how young adults can be encouraged, supported and facilitated to make healthier lifestyle changes. By adopting a more holistic approach, it should become clearer if young adults are perhaps underplaying, forgetting about, not realising or unwilling to accept the contribution that all three lifestyle behaviours make to the maintenance of a healthy lifestyle.

## Abbreviations

UK: United Kingdom.
